# The Opium Habit and Alcoholism

**Published:** 1882-02

**Authors:** 


### BIBLIOGRAPHICAL.

---

*The Opium Habit and Alcoholism.*—A treatise on the habits of Opium and its Compounds: Alcohol; Chloral-Hydrate; Chloroform; Bromide of Potassium; and Cannabis Indica; including their therapeutical indications; with suggestions for treating various painful complications. By Dr. Fred. Herman

Hubbard. Publishers: A. S. Barnes & Co., 111 & 113 William St., New York.

The rapid spread and pernicious effects of the opium habit, alcohol, &c., among the people of this country is now engaging the attention of the medical profession and other scientists to a greater degree than ever before, and the object of this well written work which treats this important subject in a comprehensive manner, is to rescue those who have become physically and mentally wrecks, and to warn all others of the danger of seductive drugs which may be innocently taken to alleviate pain, but which bring ruin on countless victims by confirmed use and stealthy habit. This work is well worthy perusal of all classes of people, and cannot fail to do good.

